# Catechin Photolysis Suppression by Aluminum Chloride under Alkaline Conditions and Assessment with Liquid Chromatography–Mass Spectrometry

**DOI:** 10.3390/molecules25245985

**Published:** 2020-12-17

**Authors:** Meei-Ju Yang, Shwu-Yuan Lee, Chieh-I Liu, Shih-Hsuan Chen, Iou-Zen Chen, Tsung-Chen Su, Jeu-Ming P. Yuann, Chien-Wei Cheng, Shiuh-Tsuen Huang, Ji-Yuan Liang

**Affiliations:** 1 Tea Research and Extension Station, Taoyuan 32654, Taiwan; 762204@gmail.com (M.-J.Y.); tres201@ttes.gov.tw (T.-C.S.); 2 Department of Tourism and Leisure, Hsing-Wu University, New Taipei City 24452, Taiwan; 091007@mail.hwu.edu.tw; 3 Department of Biotechnology, Ming-Chuan University, Gui-Shan 33343, Taiwan; allison55858@gmail.com (C.-I.L.); glory6927@gmail.com (S.-H.C.); jyuann@mail.mcu.edu.tw (J.-M.P.Y.); ochien@gmail.com (C.-W.C.); 4 Department of Horticulture and Landscape Architecture, National Taiwan University, Taipei 10617, Taiwan; chenyo@ntu.edu.tw; 5 Department of Science Education and Application, National Taichung University of Education, Taichung 40306, Taiwan; 6 Department of Soil and Environmental Sciences, National Chung Hsing University, Taichung 40200, Taiwan

**Keywords:** aluminum chloride, blue light, catechin, proanthocyanidin, superoxide anion radical

## Abstract

Tea is rich in catechins and aluminum. In this study, the process of catechin photolysis was applied as a model for examining the effects of aluminum chloride (AlCl_3_) on the structural changes of catechin and the alteration of aluminum complexes under blue light irradiation (BLI) at pH 8 using liquid chromatography and mass spectrometry techniques. Additionally, the effects of anions on catechin upon the addition of AlCl_3_ and treatment with BLI were also studied. In this study, when 1 mM catechin was treated with BLI, a superoxide anion radical ($O_{2}^{•-}$) was generated in an air-saturated aqueous solution, in addition to forming a dimeric catechin (proanthocyanidin) via a photon-induced redox reaction. The relative percentage of catechin was found to be 59.0 and 95.7 for catechin treated with BLI and catechin upon the addition of 1 mM AlCl_3_ treated with BLI, respectively. It suggested that catechin treated with BLI could be suppressed by AlCl_3_, while AlCl_3_ did not form a complex with catechin in the photolytic system. However, under the same conditions, it was also found that the addition of AlCl_3_ inhibited the photolytic formation of $O_{2}^{•-},$ and reduced the generation of proanthocyanidin, suggesting that the disconnection of proanthocyanidin was achieved by AlCl_3_ acting as a catalyst under treatment with BLI. The influence of 1 mM fluoride ($F^{-}$) and 1 mM oxalate ($C_{2}O_{4}^{2-}$) ions on the photolysis of 1 mM catechin upon the addition of 1 mM AlCl_3_ and treatment with BLI was found to be insignificant, implying that, during the photolysis of catechin, the Al species were either neutral or negatively charged and the aluminum species did not form a complex with anions in the photolytic system. Therefore, aluminum, which is an amphoteric species, has an inherent potential to stabilize the photolysis of catechin in an alkaline conditions, while suppressing the $O_{2}^{•-}$ and proanthocyanidin generation via aluminum ion catalysis in the catechin/Al system under treatment with BLI.

## 1. Introduction

Phenolic compounds defined as polyhydroxylated compounds are plant secondary metabolites. With the attachment of an aromatic ring to one or more hydroxyl groups, the structure of polyphenols includes a phenolic framework associated with other functional groups [1]. Catechins, which belong to polyphenols of plants, have been most frequently observed within chocolate, grapes, and green tea; green tea leaves are particularly rich in polyphenols, i.e., they represent about 10–30% of the leaf dry weight [2]. It has been reported that the primary ingredients in tea leaves are catechins showing advantageous properties, such as anti-oxidation, anti-radiation, anti-aging, and antimicrobial activities [3,4,5].

However, instability could be one of the major drawbacks of catechins [6]. It has been shown that catechins are not stable in aqueous solutions and thus can be readily oxidized [6]. Under oxidative processes, catechins might lose hydrogen atoms, leading to oxidized quinone products and semiquinone radical species [7,8]. It has been stated that the illumination, temperature, and pH value are important factors for the non-enzymatic browning of catechin-containing products [4,9]. In addition, it was previously reported that green tea catechins were very unstable and degraded almost completely in a few minutes in alkaline solutions (pH > 8), but were quite stable in acid solutions (pH < 4) [10]. Therefore, the oxidation of catechins under different circumstances is a major issue in the tea industry.

**Figure 1:**
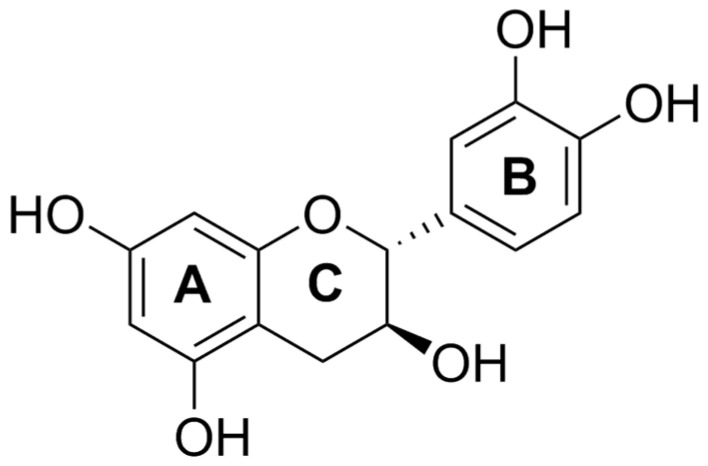
The structure of (+)-catechin.

Catechin is a flavan-3-ol with five hydroxyl groups attached to the main frame, as depicted in Figure 1. Catechin is stable in acidic conditions, while unstable under alkaline or neutral conditions during heat treatment [11]. Furthermore, catechin is unstable under ultraviolet (UV) light irradiation. Catechin and epicatechin (EC) are sensitive to UVB, as shown by the yellow photoproducts generated via UVB treatment [4]. The photolysis of catechin and EC leads to opening of the C ring of flavan-3-ols, while the epimerization of catechin can be attained via UV light irradiation [12,13]. The degradation of catechin was observed under blue light irradiation (BLI) at pH 8 [5,14]. Recently, it has been reported that dimeric catechin (proanthocyanidin) can be generated via an electron-transfer mechanism induced by catechin with BLI treatment, as evidenced by liquid chromatography and mass spectrometry, with its characteristic ion fragment being *m*/*z* 577 [5,14]. In addition, a superoxide anion radical ($O_{2}^{•-}$) can be produced upon the treatment of catechin with BLI [5,14]. The catechin or EC photolytic process can be inhibited by the addition of ascorbic acid or gallic acid during BLI, suggesting that these acids impede the electron-transfer mechanism in a photochemical reaction [5,15]. Catechin belonging to naturally occurring polyphenols is also unstable when treated with metal ions. Using spectroscopic and pulse radiolysis methods, Torreggiani et al. studied the interactions of catechin treated with Cu^2+^ or Zn^2+^ at pH 8 for 2–6 days and indicated that a great amount of yellowish auto-oxidation product was noticed via the polymerization of catechin and catechin may also act as a bidentate ligand through the catechol moiety on its B ring, thus forming a catechin–metal complex [16].

An aluminum ion (Al^3+^), which is a triply charged metal ion, possesses a relatively small ionic radius, belonging to the group of hard ions. Al is non-essential and Al^3+^ is highly reactive and toxic in biological systems [17], primarily due to its high charge density. The chemistry of Al in biological systems is dominated by its ligation to oxygen-containing functional groups mainly via substitution for other essential metal ions, with magnesium (Mg^2+^) being the most significant one [18,19]. Moreover, Al administration to animals causes neurotoxicity that is accompanied by neurofibrillary tangle formation [20].

The morphology of Al species notably varies due to the pH alteration from acidic to alkaline conditions. In aqueous solutions, Al^3+^ is amphoteric. Mujika et al. concluded that various hydrolyses of Al^3+^ species are dominant at different pHs, e.g., ${\left[\mathrm{Al}{\left(H_{2}O\right)}_{6}\right]}^{3+}$ for pH < 5.0, and ${\left[\mathrm{Al}\left(\mathrm{OH}\right){\left(H_{2}O\right)}_{5}\right]}^{2+}$, $\mathrm{trans}–{\left[\mathrm{Al}{\left(\mathrm{OH}\right)}_{2}{\left(H_{2}O\right)}_{4}\right]}^{+}$, and $\mathrm{trans}–{\left[\mathrm{Al}{\left(\mathrm{OH}\right)}_{3}{\left(H_{2}O\right)}_{3}\right]}^{0}$ for pH levels ranging from 5 to 6.2 [21]. The interaction of catechin treated with aluminum may generate Al–catechin complexes in acidic conditions. Chen et al. reported catechin in the presence of aluminum chloride (AlCl_3_) at pH 5.5 when aged for 30 days, and chemical analyses and spectroscopic studies indicated that Al reacts with the B ring of catechin, forming a 1:1-type complex [22]. Tang et al., after analyzing UV–vis spectral data, inferred that the stoichiometric ratio of Al^3+^ to catechin exceeds 2 and the polymerization of Al–catechin complexes begins at pH 6.2, suggesting that Al–catechin complex formation might decrease aluminum absorption during tea intake [23].

The Al species can be $\mathrm{trans}–{\left[\mathrm{Al}{\left(\mathrm{OH}\right)}_{3}{\left(H_{2}O\right)}_{3}\right]}^{0}$ and/or ${\left[\mathrm{Al}{\left(\mathrm{OH}\right)}_{4}\right]}^{-},\;$which are intermediates in weak alkaline solutions [21]. Previous studies show that the primary aluminum hydroxide species at the pH of the human intestine is predominately soluble ${\left[\mathrm{Al}{\left(\mathrm{OH}\right)}_{4}\right]}^{-}$ that is formed in the absence of other ligands [24,25]. According to a Geochem model employed to calculate the chemical components of green tea infusions, Liang et al. reported that the Al species at pH 7.5 are 85.16% solid with ${\mathrm{OH}}^{-}$ [Al(OH)_3_] [26]. Catechin is not stable in alkaline conditions treated with BLI [5,14]. It is therefore of interest to use catechin photolysis as a model for investigating the effects of AlCl_3_ on the changes of catechin and the alternation of aluminum under treatment with BLI in alkaline conditions. It is also crucial to inspect the mechanisms of catechin photolysis in the presence of AlCl_3_ with or without BLI treatment. Whether the coordination of complex formation is either a peroxidation reaction or a proton donation remains to be investigated.

Ferric ions (Fe^3+^) are similar to Al^3+^ in terms of their positive charges and ionic radii, with values of 60 and 53 pm, respectively [27]. The chelation of Fe^3+^ by catechin leads to three types of ferric complexes [28]. In aqueous solutions, however, Al^3+^ is amphoteric, while Fe^3+^ is not. It is therefore of interest to compare the effects of ferric chloride (FeCl_3_) and AlCl_3_ on catechin treated with BLI, as carried out in this study.

An aluminum ion (Al^3+^) can be complexed with anionic ligands, e.g., oxalate, phosphate, fluoride ($F^{-}$), and so forth, which are also known to influence the toxicity caused by Al [29]. Tea leaves accumulate ions, such as aluminum, fluorine (F), and oxalate (Ox) [30,31]. Liang et al. reported that the aluminum species in green tea infusions are primarily oxalate–Al species, while oxalate–Al and fluoro–Al are the major aluminum species in green tea infusions at pH 2.0, as shown by an ion chromatography method [32]. For solutions of catechin in the presence of AlCl_3_ with either NaF or oxalic acid under treatment with BLI in alkaline conditions, Al^3+^ might be complexed with anions, with the polymerization of catechin being achieved via photolysis and BLI treatment. It would be of interest to examine the influence exerted by fluoride ($F^{-}$) and oxalate ($C_{2}O_{4}^{2-}$) ions on the photolysis of catechin upon the addition of AlCl_3_ and treatment with BLI in alkaline conditions.

Collectively called reactive oxygen species (ROS), comprising hydrogen peroxide, hydroxyl radicals, peroxyl radicals, and superoxide anion radicals ($O_{2}^{•-}$), ROS are highly reactive in general [5,11]. Previous results show that $O_{2}^{•-}$ can be produced from catechin treated with BLI, which simultaneously inactivates *Acinetobacter baumannii* (*A. baumanni*) [5]. Catechin is an antioxidant, while $O_{2}^{•-}$ can be generated by the treatment of catechin with BLI [14]. Given the toxicity caused by ROS in biological systems, it is of critical importance to investigate the production of $O_{2}^{•-}$ that leads to structural changes of catechin, due to light excitation, upon the addition of AlCl_3_ and treatment with BLI.

For the gallic acid ester that occurs at the C_3_ position of catechins, two major types of catechins comprise about 80% of the polyphenolic compounds in green tea, with one belonging to the non-gallate-type catechins (catechin and epicatechin) and the others being gallate-type ones (epicatechin gallate (ECG) and epigallocatechin gallate (EGCG)) [33]. The compound (+)-catechin, which was used throughout this study, was purchased from a supplier, instead of being extracted from tea leaves, mainly owing to its commercial availability. Moreover, additional steps of the extraction, isolation, and purification of monomeric (+)-catechin from its numerous structural analogs and even multimers within tea leaves can be inefficient from research perspectives.

Catechin is unstable in alkaline conditions under treatment with BLI [4,12]. Aluminum ions are highly reactive in biological systems. The process of catechin photolysis was used as a model for investigating the influences of AlCl_3_ on the transformation of catechin and change of aluminum species under treatment with BLI in this study. In addition, the effects of $F^{-}$ (or oxalate) on catechin upon the addition of AlCl_3_ and treatment with BLI were also studied. Specifically, the detection of $O_{2}^{•-}$ and catechin dimer formation were used as indicators to examine the efficiency of AlCl_3_ in the photolysis of catechin.

## 2. Results

### 2.1. Effects of AlCl_3_ on Catechin Photolysis

**Figure 2:**
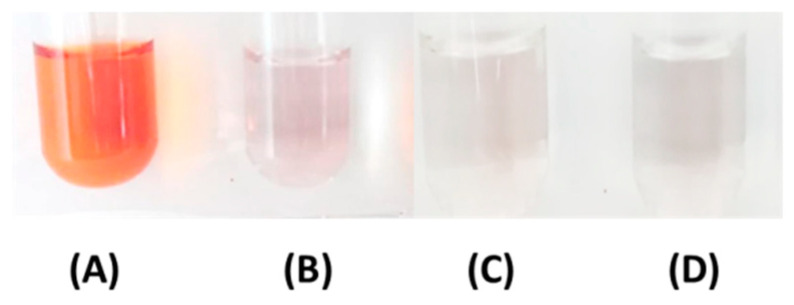
Color changes of 1 mM catechin treated (**A**) without aluminum chloride (AlCl_3_) and (**B**) with 1 mM AlCl_3_ under blue light irradiation (BLI) at 20 W/m^2^ for 120 min, and 1 mM catechin treated (**C**) without AlCl_3_ and (**D**) with 1 mM AlCl_3_ in the dark.

The effects of AlCl_3_ on catechin based on the color and spectral changes upon photolysis were investigated. As shown in Figure 2A, 1 mM catechin treated with BLI at 20 W/m^2^ for 120 min became reddish and this implied that the color alteration of catechin aqueous solution was caused by a photo-chemical reaction, while catechin irradiated with green and red lights displayed insignificant changes in color, as shown previously [14]. In Figure 2B, when 1 mM catechin was treated with BLI in the presence of 1 mM of AlCl_3_, the solution turned transparent. It has been reported that 1 mM catechin upon the addition of 0.05 mM ascorbic acid treated with BLI at 20 W/m^2^ for 120 min led to insignificant changes, suggesting that ascorbic acid can suppress the photosensitive oxidation of catechin via its strong reducing power [5].

**Figure 3:**
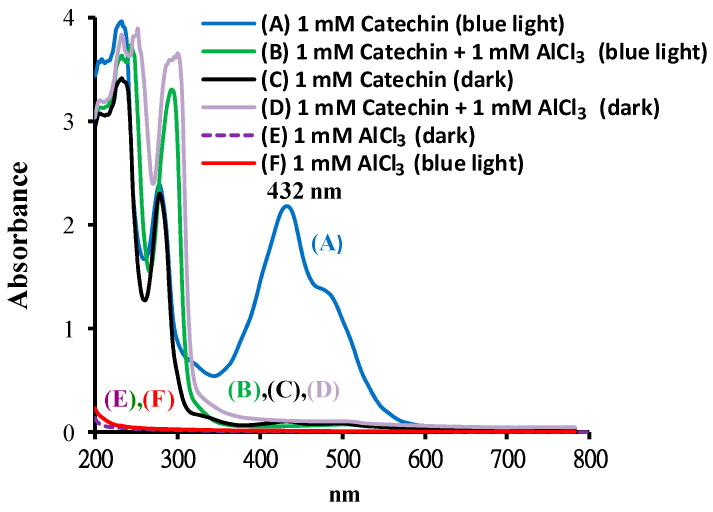
The absorption spectra of 1 mM catechin treated (A) without and (B) with 1 mM AlCl_3_ under BLI at 20 W/m^2^ for 120 min, and those of 1 mM catechin treated (C) without and (D) with 1 mM AlCl_3_ in the dark. The absorption spectra of the solution containing 1 mM AlCl_3_ treated (E) without or (F) with BLI at 20 W/m^2^ for 120 min. The wavelengths for scanning reaction solutions were set in the region of 200–800 nm.

Figure 3 shows the spectra of catechin treated with or without AlCl_3_ at pH 8 after BLI. The results indicate that the catechin solution exhibited two absorption peaks at 232 and 278 nm, as shown in Figure 3(C) (black line), while, for the catechin treated with BLI, there were three absorption peaks noticed at 232, 278, and 432 nm (Figure 3(A), blue line). The area under the curve peak at 432 nm was significantly increased by catechin treated with BLI. The absorption spectrum of 1 mM AlCl_3_ per se under BLI is shown in Figure 3(F) (red line). In Figure 3(B), by adding 1 mM AlCl_3_, the BLI-treated catechin exhibited an almost complete disappearance in the area under the curve peak at 432 nm, in addition to enhanced absorption appearing in the UV region. The enhanced UV absorption of samples in the presence of AlCl_3_ is likely due the ligand-to-metal charge transfer within Al(OH${)}_{4}^{-}$ species at an alkaline pH (Figure 3(F)) [34]. There was no significant change in the absorption bands in the visible light range for 1 mM catechin in the dark or catechin upon the addition of 1 mM AlCl_3_ under BLI treatment, suggesting that a chromogenic species was generated by a photolytic reaction of catechin, which can be diminished by AlCl_3_, as shown in the current study.

### 2.2. HPLC–MS Assay of Catechin upon the Addition of AlCl_3_ Treated with BLI

**Figure 4:**
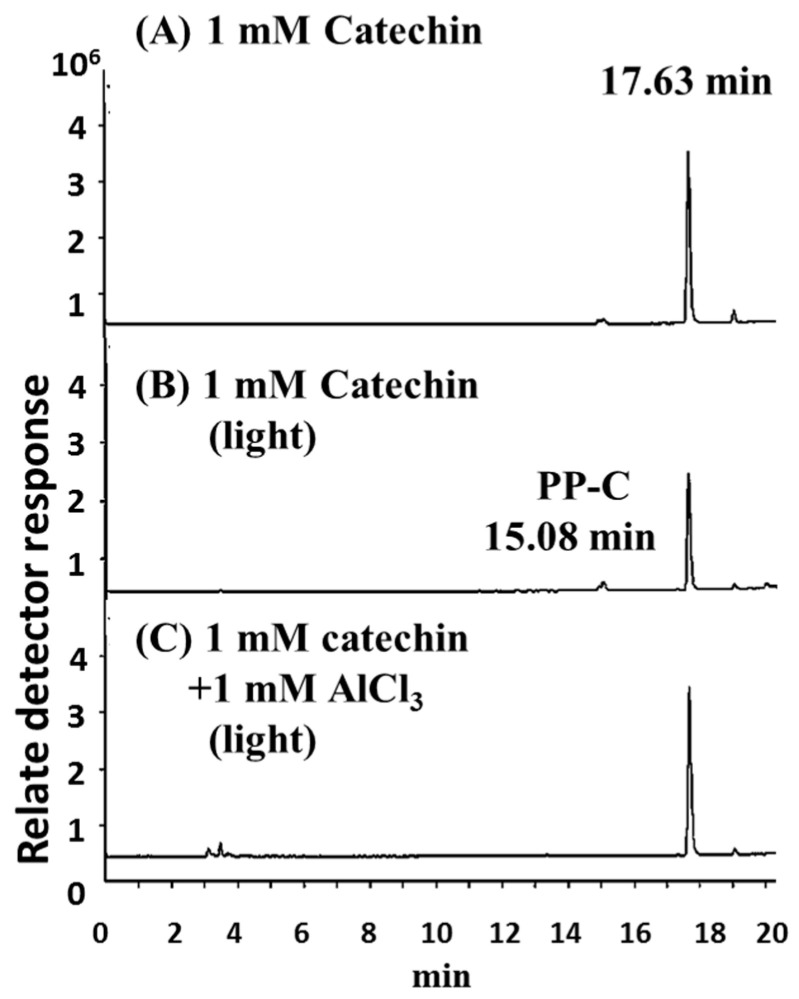
Total ion chromatograms of HPLC–MS analyses of (**A**) 1 mM catechin in the dark and 1 mM catechin in the absence (**B**) or presence (**C**) of 1 mM AlCl_3_ treated with BLI at 20 W/m^2^ for 120 min at pH 8. PP-C: The photoreaction product from the catechin.

**Figure 5:**
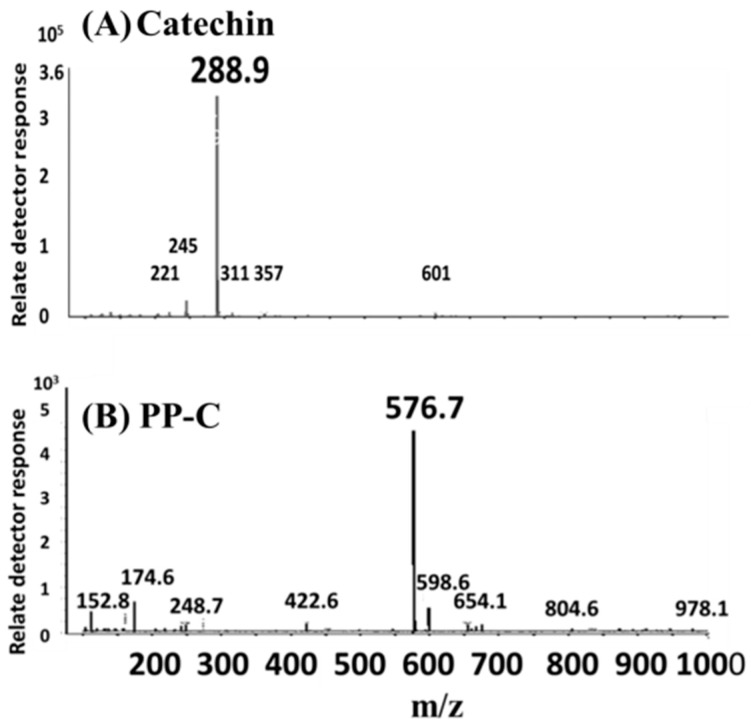
Electrospray ionization mass spectrum of (**A**) catechin at 17.63 min and (**B**) the photoreaction product from the catechin (PP-C) at 15.08 min.

Analyses of catechin and its polymerization were undertaken in this study. The total ion chromatograms obtained by HPLC–MS analysis for catechin treated with or without AlCl_3_ at pH 8 after BLI at 20 W/m^2^ for 120 min are shown in Figure 4. From Figure 4A, the total ion chromatogram of the catechin solution (pH 8) was established at 17.63 min. Hence, catechin was eluted at 17.63 min and verified by its mass spectrum, with the fragmented ion species appearing at *m*/*z* 288.9, as shown in Figure 5A. In Figure 4B, it can be observed that the total ion chromatogram of catechin was significantly reduced and the photoreaction product from the catechin (PP-C) at 15.08 min was generated after the solution containing catechin was treated with BLI. The PP-C could be identified at 15.08 min, with its characteristic ion fragment being *m*/*z* 576.7, as shown in Figure 5B. The molecular weight of catechin and the PP-C were 290 and 578 g/mol, respectively.

According to a previous report, it was proposed that PP-C is a catechin dimer, which is a dimeric B-type proanthocyanidin [5,14]. The total ion chromatogram of blue light-treated 1 mM catechin upon the addition of 1 mM AlCl_3_ at pH 8 exhibited a major elution peak at 17.63 min, as shown in Figure 4C. The catechin in the solution eluted at 17.63 min was verified by its MS spectrum, and a major ion peak was observed at *m*/*z* 288.9. Mass spectroscopic signals were determined according to the quasimolecular anions [M − H]^−^.

**Figure 6:**
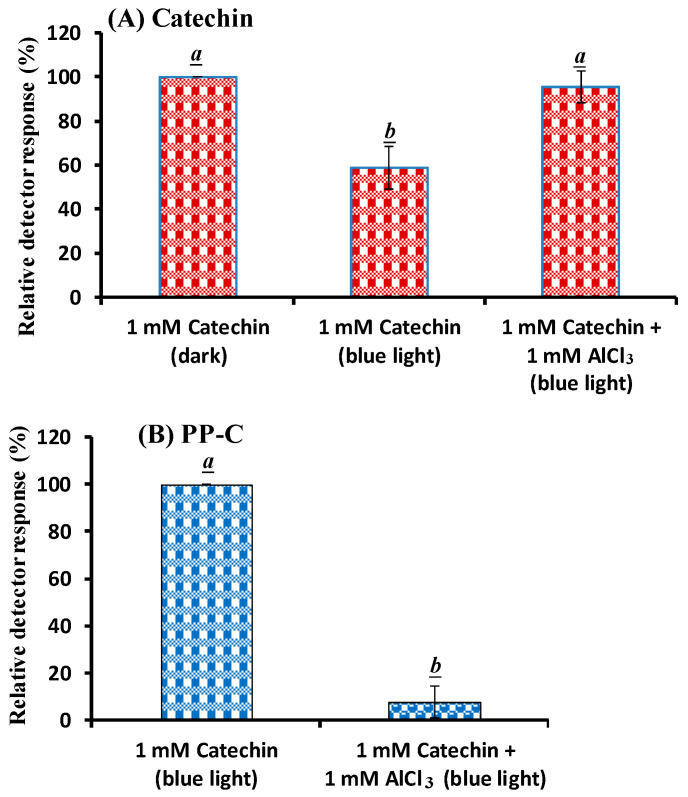
The relative components of (**A**) catechin and (**B**) proanthocyanidin remaining after 1 mM catechin was treated with or without 1 mM AlCl_3_ under BLI at 20 W/m^2^ for 120 min. PP-C: The photoreaction product from the catechin. Statistical differences between groups are denoted by different letters (a and b) above each bar. The level set for statistical significance was *p*-value < 0.05.

The different levels of catechin and catechin with AlCl_3_ treated with BLI at 20 W/m^2^ for 120 min were obtained by the relative percentage of the peaks from the HPLC–MS assay. The relative percentage of catechin was found to be 59.0 and 95.7 for 1 mM catechin and 1 mM catechin with 1 mM AlCl_3_ treated with BLI, respectively (Figure 6A). The relative percentage of the catechin dimer (proanthocyanidin) was found to be 100 and 7.68 for catechin and catechin with 1 mM AlCl_3_ treated with BLI, respectively (Figure 6B). The results indicate that catechin treated with BLI could be suppressed by AlCl_3_.

### 2.3. Effects of FeCl_3_ on Catechin Treated with BLI

**Figure 7:**
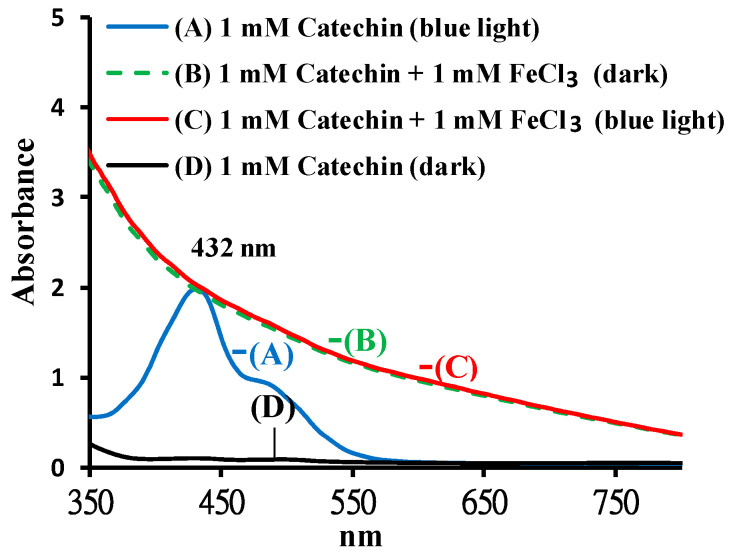
The absorption spectra of 1 mM catechin upon the addition of 1 mM ferric chloride (FeCl_3_) treated with or without BLI at 20 W/m^2^ for 120 min at pH 8.

The effects of FeCl_3_ on catechin upon photolysis were investigated based on the spectral changes. The spectra of catechin treated with or without FeCl_3_ at pH 8 under BLI at 20 W/m^2^ for 120 min are shown in Figure 7. In the solution of catechin, two absorption peaks were observed at 232 and 278 nm, as shown in Figure 3(C). In Figure 3(A), for catechin treated with BLI, three absorption peaks were noticed at 232, 278, and 432 nm, while, for catechin treated with or without BLI in the presence of 1 mM FeCl_3_ (pH 8), their distinct curves were noticed in the respective absorption spectra (Figure 7), very unlike catechin treated with BLI alone. This suggests that treatment with BLI at 20 W/m^2^ for 120 min has little effect on catechin in the presence of 1 mM FeCl_3._ As for the spectra between 200 and 350 nm, the samples of catechin treated with or without BLI in the presence of FeCl_3_ (Figure 7) exhibited absorption well beyond the detection limits of the instrument. However, after a ten-fold dilution, their whole absorption profiles remained essentially the same (data not shown).

### 2.4. Effects of Sodium Fluoride (NaF) or Oxalic Acid on Catechin upon the Addition of AlCl_3_ Treated with BLI

**Figure 8:**
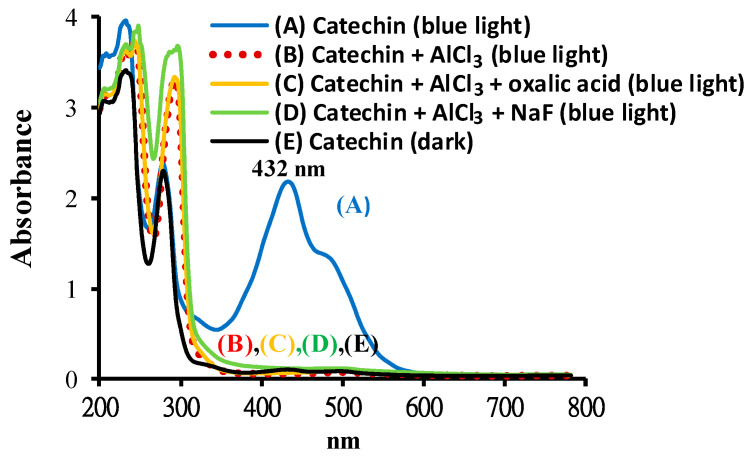
The absorption spectra of 1 mM catechin treated (A) without AlCl_3_, (B) with 1 mM AlCl_3_, (C) with 1 mM AlCl_3_ upon the addition of 1 mM oxalic acid, (D) with 1 mM AlCl_3_ upon the addition of 1 mM sodium fluoride (NaF) treated with BLI at 20 W/m^2^ for 120 min at pH 8 and (E) in the dark. The wavelengths for scanning the reaction solutions were set in the region of 200–800 nm.

The effects of NaF or oxalic acid on catechin in the presence of AlCl_3_ treated with BLI at 20 W/m^2^ for 120 min were investigated. As shown in Figure 2D, the catechin treated with BLI at 20 W/m^2^ for 120 min became reddish in color. For catechin in the solution, the absorption peak at 432 nm was significantly increased following BLI. There were three absorption bands at 232, 278, and 432 nm exhibited by catechin solution treated with BLI, as shown in Figure 8(A). For solutions of catechin in 1 mM in the presence of AlCl_3_ with either 1 mM NaF or 1 mM oxalic acid under treatment with BLI, the reaction solution became transparent and the absorption band only appeared in the UV light range, which was attributed to the absence of catechin absorption bands in visible light, as shown in Figure 8(B)–(D). There was no significant change in the absorption bands in the visible light range for catechin in the dark and catechin in 1 mM in the presence of AlCl_3_ with either 1 mM NaF or 1 mM oxalic acid under treatment with BLI.

### 2.5. Detection of $O_{2}^{•-}$ in Catechin upon the Addition of AlCl_3_ Treated with BLI

The generation of $O_{2}^{•-}$ from catechin illuminated with blue light was studied by the nitrotetrazolium blue chloride (NBT) reduction method [5,35]. The generated $O_{2}^{•-}$ can reduce NBT to form formazan, which can be detected at 560 nm. It has also been reported that catechin hydrate or epicatechin treated with BLI at 20 W/m^2^ for 0–60 min generates $O_{2}^{•-}$ by the NBT reduction method [5,15]. The effects of AlCl_3_ on the generation of $O_{2}^{•-}$ from the photolytic process of catechin were investigated in this study.

**Figure 9:**
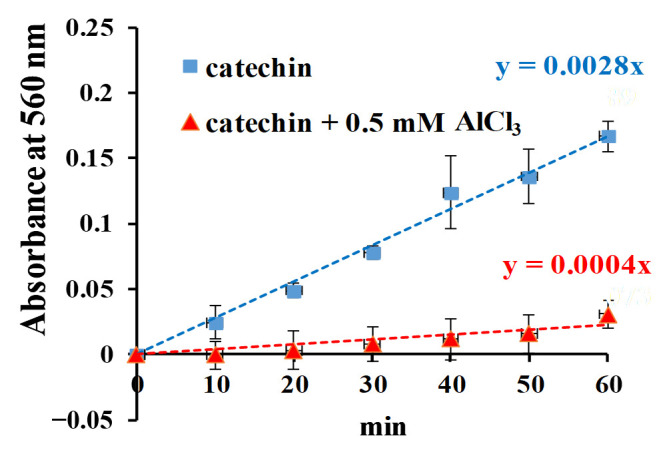
Effects of 1 mM catechin or 1 mM catechin upon the addition of 0.5 mM AlCl_3_ on nitrotetrazolium blue chloride (NBT) reduction under BLI at 20 W/m^2^ for 0–60 min.

As shown in Figure 9, the photochemical effect of the NBT reduction in catechin was increased in a time-dependent manner of 0–60 min under BLI at 20 W/m^2^, suggesting that $O_{2}^{•-}$ was produced from catechin under BLI at a pH of 7.8 in this study. The slopes of the NBT reaction curve of the $O_{2}^{•-}$ production for catechin and catechin with AlCl_3_ at the same conditions were 0.0028 and 0.0004, respectively, as shown in Figure 9, suggesting that AlCl_3_ might inhibit $O_{2}^{•-}$ generation via the photolytic process of catechin in the catechin/NBT system.

## 3. Discussion

The effects of AlCl_3_ on the photooxidation and polymerization of catechin and the alteration of aluminum complexes in the catechin/Al system treated with BLI in alkaline conditions were examined in this study.

**Figure 10:**
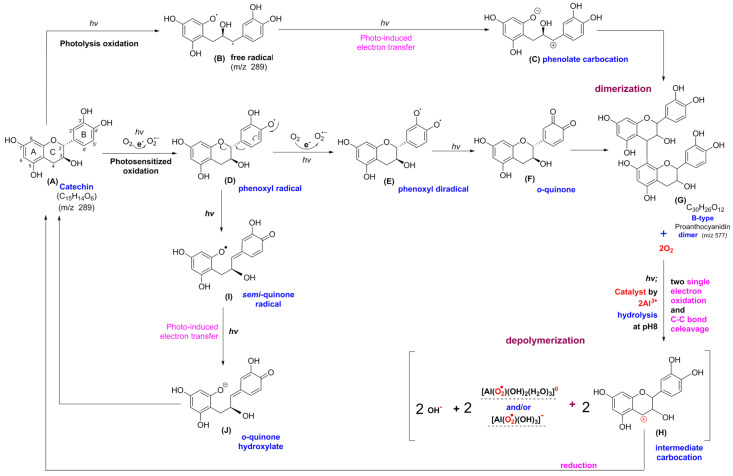
Proposed mechanism for the photolytic reaction of catechin upon the addition of AlCl_3_. (**A**): catechin, (**B**): free radicals at *m*/*z* 289, (**C**): phenolate carbocation, (**D**): phenoxyl radical, (**E**): phenoxyl diradical, (**F**): *o*-quinone at *m*/*z* 287, (**G**): B-type proanthocyanidin, (**H**): catechin carbocation, (**I**): *semi*-quinone radical, and (**J**): *o*-quinone hydroxylate.

Catechin treated with BLI under alkaline conditions was able generate a chromogenic catechin dimer, as shown in Figure 2 and Figure 3. In addition, under BLI and alkaline conditions, catechin generated $O_{2}^{•-}$ by photosensitize oxidation via a photo-oxidative process (Figure 9), and a B-type proanthocyanidin was formed and identified by its characteristic ion fragment of *m*/*z* 576.7 due to a condensation reaction, as shown in Figure 4, Figure 5 and Figure 6. Our previous study showed that a B-type proanthocyanidin was generated from the photooxidation of catechin upon treatment with BLI [5]. Based on the results of this study, we propose a catechin photolytic reaction mechanism under the influence of AlCl_3_ and under alkaline conditions, as shown in Figure 10. In our previous study, two pathways were proposed regarding the photooxidation of catechin upon treatment with BLI [5]. Wilhelm-Mouton et al. examined the catechin and epicatechin irradiated by UV in MeOH and found the structural change of the catechin molecule which was carried out to open the heterocyclic ring (C ring) of flavan-3-ols via photolytic cleavage of the ether bond [13]. Shi et al. also reported that the catechin/epicatechin molecule was excited at two positions under UV irradiation, i.e., the –OH bond and heterocyclic ring. Excitation of the –OH bond led to the generation of a quinone compound, while the heterocyclic ring of catechin/epicatechin was preferentially opened via photolytic cleavage at the C–O ether linkage with low bond dissociation energies, which resulted in the generation of free radicals at *m*/*z* 289 [4]. As shown in Figure 10B, the heterocyclic ring C of catechin was photolytically and exclusively broken via cleavage of the ether bond, achieved by low bond dissociation energies, with a free radical species being generated at *m*/*z* 289 [4,13]. The radical species thus formed could be further ionized in a polar solvent via an electron transfer process to produce a transient catechin phenolate carbocation, as shown in Figure 10C [36]. Catechin oxidation caused by BLI involves the loss of an electron from its B ring, accompanied by the generation of $O_{2}^{•-}$ from molecular oxygen. The –OH bond of the B ring was excited via photosensitive oxidation by forming a quinone compound observed at *m*/*z* 287, as shown in Figure 10F. It has also been reported that the quinone compound formed during the process as the primary product is unstable and may undergo further reactions. Additionally, the quinone compound can spontaneously combine with a catechin carbocation due to their high affinity, and in the process, the resultant dimer or polymer may rearrange their structures through an enol-like conversion reaction by trapping two hydrogen atoms and form new diphenols [37]. Ojwang et al. investigated the proanthocyanidin composition and detected the major MS/MS fragment ions *m*/*z* 287 and 289, derived from B-type proanthocyanidin at *m*/*z* 577, suggesting that the compound is a 4→8 linked procyanidin dimer, according to the analyses by liquid chromatography and mass spectrometry [38]. Moreover, in Figure 10G, a B-type proanthocyanidin was noticed, formed by an additional proton transfer and condensation of the quinone compound with the catechin phenolate carbocation intermediate [5,38,39,40]. Simultaneously, the semi-quinone radical was quenched by photo-induced electron transfer, forming an *o*-quinone intermediate, as shown in Figure 10I,J [5]. Then, the conversion of compounds, from Figure 10J to Figure 10A, was achieved via cyclization and protonation.

Phenolic compounds containing aromatic hydroxyl groups can form complex compounds with metal ions [41]. Kumamoto et al. applied an apparent acid dissociation constant (p*K*_a1_) to examine the binding of metal ions to epicatechin. The p*K*_a1_ of epicatechin was measured in the presence of Al^3+^, Cu^2+^, Fe^2+^, and Fe^3+^. The results showed that the p*K*_a1_ of epicatechin (8.68) was significantly decreased by Cu^2+^ (6.84), Fe^2+^ (6.28), and Fe^3+^ (6.33), but was only slightly changed when Al^3+^ (8.33) was present, suggesting that Cu^2+^, Fe^2+^, and Fe^3+^ preferentially bind to epicatechin [42]. The color of 1 mM catechin upon the addition of 1 mM FeCl_3_ treated with or without BLI turned black in this study and exhibited distinct degrees of absorption spectra of catechin under BLI, as shown in Figure 7. As shown in Figure 6A, the average relative percentage of catechin was found to be 100 and 95.7 for catechin in the dark and catechin treated with BLI in the presence of 1 mM AlCl_3_ at pH 8, respectively, with no statistically significant difference (*p* > 0.05) being noticed between these two treatments. Despite the fact that both aluminum and ferric ions bear the same positive charge (3+), the effects of AlCl_3_ and FeCl_3_ on the photooxidation stability of catechin treated with BLI at pH 8 were found to be quite different, according to this study. 

Al^3+^ is reactive, primarily due to its high charge density. Torreggiani et al. reported that catechin, when treated with Cu^2+^ or Zn^2+^ at pH 8, acts as a bidentate ligand through the catechol moiety on the B ring in the catechin–metal complex reaction [16]. Tang et al. suggested that the polymerization of Al–catechin can occur via the hydroxyl group of the C ring of catechin connected with Al^3+^ by chelation at pH 6.2, and that the Al–catechin complex might reduce aluminum absorption in the intestine [23]. As such, the generation of an Al–catechin complex likely significantly decreases the content of catechin. In this study, the average relative percentage of catechin was found to be 100 and 94.9 for catechin and catechin treated with 1 mM AlCl_3_ in the dark at pH 8 based on the HPLC–MS assay, respectively, and no statistically significant difference (*p* > 0.05) was noticed between these two treatments. In addition, it was observed that the spectroscopic changes of catechin in the dark and catechin upon the addition of AlCl_3_ treated with or without $F^{-}$ ($C_{2}O_{4}^{2-}$) under BLI were not significant, as shown in Figure 8. AlCl_3_ is an amphoteric compound, as the Al species can be $\mathrm{trans}-{\left[\mathrm{Al}{\left(\mathrm{OH}\right)}_{3}{\left(H_{2}O\right)}_{3}\right]}^{0}$ and/or ${\left[\mathrm{Al}{\left(\mathrm{OH}\right)}_{4}\right]}^{-},\;\mathrm{which}\;$are the intermediates in a weak alkaline solution [21]. Both $F^{-}$ and $C_{2}O_{4}^{2-}$ had little influence on AlCl_3_ upon the addition of catechin under BLI in this study, implying that the aluminum hydroxide species in catechin photolysis at pH 8 was either neutral or negatively charged, and that the aluminum hydroxide species does not react with catechin to generate an Al–catechin complex at pH 8, as observed in this study. 

Under BLI and alkaline conditions, the photooxidation of catechin occurred and generated proanthocyanidin and $O_{2}^{•-}$, but both proanthocyanidin and $O_{2}^{•-}$ were reduced by catechin upon the addition of AlCl_3_ during the photolytic process, as shown in Figure 2, Figure 3 and Figure 4, Figure 6 and Figure 9. When catechin was treated with BLI, the percentage of catechin was decreased by 41.0, while between catechin left in the dark and that treated with BLI in the presence of AlCl_3_, the difference of the average catechin content was statistically insignificant (*p* > 0.05), as shown in Figure 6A. Based on the results of this study, we can explain the catechin photolytic reaction under the influence of AlCl_3_ and under alkaline conditions as follows.

Exley reported that $O_{2}^{•-}$ bound to Al^3+^ forms a putative aluminum superoxide semi-reduced radical ion—${\left({\mathrm{Al}}^{3+}O_{2}^{•}\right)}_{\;}^{-}$—by which the complex not only explains the pro-oxidant activity of aluminum, but also connects the catalytic activity of the non-redox-active Al^3+^ with superoxide-driven oxidation, as shown in Equation (1) [43]:

(1) $$O_{2}^{•-}+{\;\mathrm{Al}}^{3+}\;\overset{\;}{↔}\;{\left({\mathrm{Al}}^{3+}O_{2}^{•}\right)}_{\;}^{-}$$

Mujika et al. indicated that the formation analysis of an aluminum superoxide complex in aqueous solution should take into account the capacity of superoxide to displace one of the water/hydroxide ligands within the first coordination shell surrounding the Al^3+^ cation [21]. According to the free energy calculation regarding the displacement of water/hydroxide ligands by superoxide, the ${\mathrm{Al}}^{3+}$ superoxide complex was formed via displacement of a water molecule from the first solvation shell of ${\mathrm{Al}}^{3+}$ in an aqueous solution [21]. Mujika et al. also reported the Al^3+^ superoxide complex formation is pH-dependent in aqueous solutions, as aluminum shows an intrinsic high potential to stabilize a superoxide anion as a linear relationship of interactions between a metal ion and a superoxide anion is established [21]. The Al species can be $\mathrm{trans}–{\left[\mathrm{Al}{\left(\mathrm{OH}\right)}_{3}{\left(H_{2}O\right)}_{3}\right]}^{0}$ and/or ${\left[\mathrm{Al}{\left(\mathrm{OH}\right)}_{4}\right]}^{-},\;$which are intermediates in weak alkaline solutions, and the equations for the substitution reactions of one ${\mathrm{OH}}^{-}/H_{2}O$ ligand by $O_{2}^{•-}$ are shown in Equations (2) and (3) [21]:

(2) $$\begin{array}{c} {\left[\mathrm{Al}{\left(\mathrm{OH}\right)}_{3}{\left(H_{2}O\right)}_{3}\right]}^{0}+{\;O}_{2}^{•-}\;\to \;{\left[\mathrm{Al}\left({\;O}_{2}^{•}\right){\left(\mathrm{OH}\right)}_{3}{\left(H_{2}O\right)}_{2}\right]}^{-}+{\;H}_{2}O\\ \to \;{\left[\mathrm{Al}\left({\;O}_{2}^{•}\right){\left(\mathrm{OH}\right)}_{2}{\left(H_{2}O\right)}_{3}\right]}^{0}+{\mathrm{OH}}^{-} \end{array}$$

(3) $${\left[\mathrm{Al}{\left(\mathrm{OH}\right)}_{4}\right]}^{-}+{\;O}_{2}^{•-}\;\to \;{\left[\mathrm{Al}\left({\;O}_{2}^{•}\right){\left(\mathrm{OH}\right)}_{3}\right]}^{-}+{\mathrm{OH}}^{-}$$

Redox-inactive metal ions can both act as a Lewis acid and play a pivotal role in promoting various reactions [44]. Previous studies have shown that the binding of ionic metal species with radical anions plays an important role in mediating the electron-transfer reactivity of the substrate [45]. The complexation of $O_{2}^{•-}$ to metal ions can be achieved by photo-induced electron transfer from the excited state of a dimeric organic compound, in which the catalytic process acts as a unique two-electron donor and then yields two monomeric cations along with two $O_{2}^{•-}$–metal ion complex radical ions [44,46]. Mujika et al. reported the finding that ${\mathrm{Al}}^{3+}$ superoxide complex formation can promote the effects of electron-transfer processes, while leading to important pro-oxidant activity through a superoxide formation mechanism and the generation of an ${\mathrm{Al}}^{3+}$ superoxide complex, suggesting a plausible pathway of aluminum-mediated oxidative catalysis [21]. 

Achilonu et al. reported that the procyanidin B-3-type dimer can be generated via the oxidative formation of tetra-*O*-methyl-3-oxo-catechin [47]. The intermediates of the tetra-*O*-methyl-3-oxo-catechin radical cation and the tetra-*O*-methyl-3-oxo-carbocation can form through single electron transfer by Ag^+^ acting as a catalyst in the process of synthesizing procyanidin B-3-type dimer derivatives [47]. Ma et al. also reported that proanthocyanidin can be cleaved into a flavan-3-ol C-4 carbocation intermediate and flavan-3-ol in diluted mineral acids [48]. In this study, the disconnection of the dimeric structure of a B-type proanthocyanidin (BtPA) could be achieved by Al^3+^ acting as a catalyst under treatment with BLI. The BtPA was oxidized by outer-sphere electron transfer, forming the semi-oxidized (BtPA^•^)^+^ and the semi-reduced ${\left(O_{2}^{•}\cdots {\mathrm{Al}}^{3+}\right)}^{-}$ radical species simultaneously in Equation (4). Then, C-4 carbocation of catechin and ${\mathrm{catechin}}^{•+}\;$were produced by the C–C bond cleavage in an additional proton transfer process in Equation (5). The ${\mathrm{catechin}}^{•+}$, which is an A-ring radical cation, generates the resonance-stabilized benzylic radical species via losing a proton in Equation (6). The benzylic intermediate carbocation and ${\left(O_{2}^{•}\cdots {\mathrm{Al}}^{3+}\right)}^{-}\;$radical anion were generated via Al^3+^ catalysis, followed by secondary electron oxidation, as shown in Equation (7). The depolymerization of BtPA via photooxidation in total chemical reactions by aluminum ion catalysis is represented in Equation (8) [46,49]. Subsequently, the carbocation (Figure 10H) can be reduced to generate catechin at *m*/*z* 289 (Figure 10A) [5,15,48].

(4) $$BtPA\;+{\;O}_{2}\;\overset{hv,\;Al^{3+}\;}{\underset{{\;e}^{-}\;\mathrm{transfer}}{⟶}}\;BtPA^{•+}+{\left(O_{2}^{\;•\;}\cdot \cdot \cdot {\mathrm{Al}}^{3+}\right)}^{-}$$

(5) $$BtPA^{•+}\overset{hv,\;H^{+}}{\underset{bond\;cleavage}{⟶}}\mathrm{carbocation}+catechin^{•+\;}$$

(6) $$catechin^{•+}\;\overset{\;hv}{\underset{-H^{+}}{\to }}\;catechin^{•\;}$$

(7) $$catechin^{•\;}+{\;O}_{2}\;\overset{hv,\;Al^{3+}\;}{\underset{{\;e}^{-}\;\mathrm{transfer}}{⟶}}\;\mathrm{carbocation}\;+{\left(O_{2}^{\;•\;}\cdot \cdot \cdot {\mathrm{Al}}^{3+}\right)}^{-}$$

Therefore, Equations (6)–(9) can be integrated into:

(8) $$BtPA\;+2{\;O}_{2}\;\overset{hv,\;2Al^{3+}\;}{\underset{\;2{\;e}^{-}\;\mathrm{transfer}}{⟶}}\;2\;\mathrm{carbocation}\;+2\;(O_{2}^{\;•-\;}\cdot \cdot \cdot {\mathrm{Al}}^{3+})$$

The formation of superoxide–Al^3+^ metal ion complexes and the electron transfer catalysis described above, where ${\mathrm{BtPA}}^{•+}\;$is a B-type proanthocyanidin radical cation, i.e., ${\left(O_{2}^{•}\cdots {\mathrm{Al}}^{3+}\right)}^{-},\;$is characteristic of an aluminum-superoxide radical anion.${\;\mathrm{Catechin}}^{•}\;$is a catechin radical and, by electron transfer catalysis, it turns into ${\mathrm{catechin}}^{+},\;$which is also called an intermediate C-4 carbocation of catechin, ${\mathrm{catechin}}^{•+},\;\mathrm{which}\;\mathrm{is}\;$a catechin radical cation. The dotted line (⋯) indicates a substitution reaction of ${\mathrm{OH}}_{2}/{\mathrm{OH}}^{-}\;$by $O_{2}^{•-}\;$at the first coordination shell of the hydrolytic ${\mathrm{Al}}^{3+}\;$species. 

## 4. Materials and Methods

### 4.1. Chemicals

Aluminum chloride (AlCl_3_), ferric chloride (FeCl_3_), methanol, oxalic acid, and sodium fluoride (NaF) were obtained from Sigma-Aldrich (St. Louis, MO, USA). (+)-Catechin was obtained from Toronto Research Chemicals Inc. (Toronto, ON, Canada). Nitrotetrazolium blue chloride (NBT) was purchased from Bio Basic, Inc. (Markham, ON, Canada). Deionized water was made by the Milli-Q system, which was used for preparing the solutions during this research. 

### 4.2. Photolytic System

**Figure 11:**
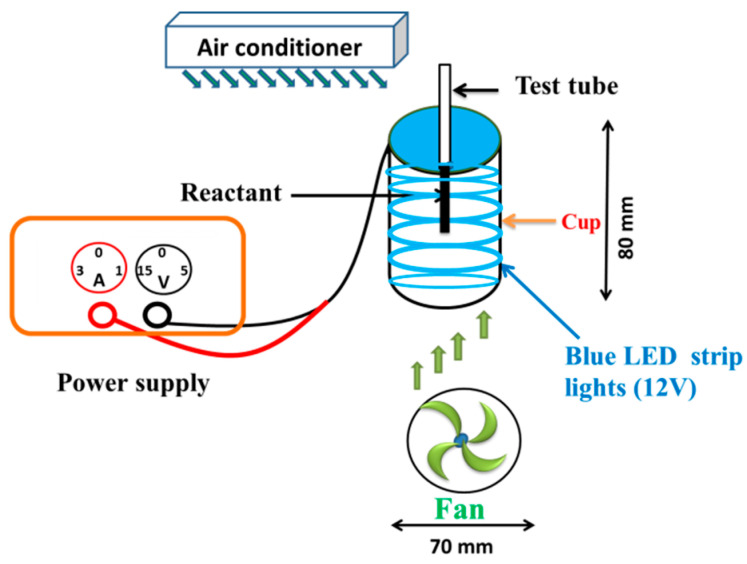
Experimental setup of the photolytic equipment.

The photolytic system was composed of a power supply and a radiation unit made of opaque plastic, as described previously [35,50]. A glass tube containing the test solution was fixed to the upper end of the photolytic system; six 5050 SMD LED strip lights (vitaLED Tech. Co., Tainan, Taiwan) were placed inside the cup. The blue light irradiance was kept at 20 W/m^2^ and maintained by a solar power meter (TM-207, Tenmars Electronics, Taipei, Taiwan), and the temperature of the photolytic system was maintained at 25 ± 3 °C, monitored by an infrared thermometer (MT 4, Raytek, CA, USA), and controlled by an air conditioner and electric fan during the irradiation experiments. The emission spectra (λ_max_ = 465 nm) of the blue light spectrum were measured using a UV–vis miniature fiber optic spectrometer (USB4000, Ocean Optics, Dunedin, FL, USA), with the peak width at half height (W_1/2_) being 26 nm. The experimental setup of the photoreaction system is shown in Figure 11.

### 4.3. The Effect of AlCl_3_ on Catechin Treated with BLI

Blue light was applied as a light source in the catechin-photosensitized reaction. In our previous study, treatment of a photoreaction involving 1 mM catechin at pH 8 inhibited the growth of *A. baumanni* by 5–6 logs under BLI at 20 W/m^2^ for 120 min [5]. Catechin is unstable in alkaline conditions under treatment with BLI [5,14]. Accordingly, we modified and controlled the pH at 8.0 in this experiment to investigate the effects of AlCl_3_ on catechin treated with BLI. Briefly, 1 mM catechin in H_2_O at pH 8 left in the dark was used as a control, while 1 mM catechin in water at pH 8 treated with BLI at 20 W/m^2^ for 120 min and catechin (1 mM) upon the addition of 1 mM AlCl_3_ treated with BLI at 20 W/m^2^ for 120 min were used for the treatment conditions. The pH of the reaction solution was kept at 8 by adding either 1 N NaOH or HCl. The reaction solutions were detected in the absorbance range of 250–750 nm via an ELISA microplate reader (Thermo Fisher Scientific Multiskan spectrophotometer, Waltham, MA, USA).

### 4.4. The Effect of FeCl_3_ on Catechin Treated with BLI

The effects of FeCl_3_ on catechin treated with BLI were also investigated in this study. Briefly, catechin (1 mM) in H_2_O at pH 8 left in the dark was utilized as a control and catechin (1 mM) treated without FeCl_3_ and with 1 mM FeCl_3_ under BLI at 20 W/m^2^ for 120 min were used for the treatment conditions. The absorbance of each reaction solution was detected by an ELISA reader in the range of 250–750 nm.

### 4.5. The Effect of NaF or Oxalic Acid on Catechin Treated with BLI in the Presence of AlCl_3_

The effects of NaF or oxalic acid on catechin upon the addition of AlCl_3_ treated with BLI were also investigated in this study. Briefly, catechin (1 mM) in H_2_O at pH 8 left in the dark was utilized as a control, while catechin (1 mM) treated with BLI, catechin (1 mM) upon the addition of 1 mM AlCl_3_ treated with BLI at 20 W/m^2^ for 120 min, catechin (1 mM) upon the addition of 1 mM AlCl_3_ and 1 mM NaF treated with BLI at 20 W/m^2^ for 120 min, and catechin (1 mM) upon the addition of 1 mM AlCl_3_ and 1 mM oxalic acid treated with BLI at 20 W/m^2^ for 120 min were used as the treatment conditions. The absorbance of each reaction solution was detected by an ELISA reader in the range of 250–750 nm.

### 4.6. HPLC–MS Assay of Catechin upon the Addition of AlCl_3_ Treated with BLI

The effects due to BLI on the changes of catechin were studied by HPLC–MS analysis, as described by previous results [5,15]. The reactants were prepared as described in Section 4.3. The samples were analyzed by the Agilent 1200 Series HPLC System and the 6410B triple quadrupole tandem MS system (Agilent Technologies, Palo Alto, CA, USA), with the electrospray ionization (ESI) technique as the ionizing source. MS analysis in this study was conducted in a negative ion mode. The parameters of the instrument were set as follows: Drying gas (N_2_) temperature and flow were 350 °C and 11 mL/min, respectively; the nebulizer gas pressure was 50 psi; and the capillary voltage and temperature were 3700 V and 280 °C, respectively. A complete scan for data ranging from 100 to 1000 atomic mass unit (amu) was conducted. Data acquisition and analyses were performed with the Agilent Mass Hunter Workstation software (version B.06.00., Agilent Technologies, Palo Alto, CA, USA).

The solutions of all of the reactions were eluted via an EC-C18 column (Poroshell 120; particle size 2.7 µm, inner diameter 4.6 mm, length 150 mm; Agilent Technologies, Palo Alto, CA, USA) at 40 °C. All of the reaction solutions were prepared as depicted in Section 4.3 and the solutions were filtered by a 0.45 μm filter (Millipore, Brighton, MA, USA) prior to each analysis. 

The separation of catechin and its photoproducts was accomplished with a mobile phase comprising both methanol as solvent A and 0.1% methanoic acid as solvent B. The gradient elution performed in this study can be described as follows: The linear gradient of solvent A was started within 0–3 min, 1–10%; 3–10 min, 10–20%; 10–16.5 min, 20–75%; 16.5–20 min, 75–50%; and 20–23 min, 50–1%. Each sample of 5 μL was injected into the chamber and the flow rate of the elution was set to 400 μL/min.

### 4.7. Assay of $O_{2}^{•-}$

Previously, the effects of the catechin hydrate photoreaction on the generation of $O_{2}^{•-}$ were investigated [5]. The NBT reduction method was applied to evidence $O_{2}^{•-}$ production, modified from the method of the riboflavin photoreaction, as described earlier [51]. The influence of AlCl_3_ on $O_{2}^{•-}$ generation in the catechin photolytic process was therefore evaluated by the NBT reduction method. All of the fresh reaction solutions were ready prior to each experiment. Firstly, 0.11 g L-methionine was added to 73.3 mL of deionized water. Then, 10 mg NBT powder and 8 mL 10 mM catechin (treated with or without 5 mM AlCl_3_) were added to the solution. The final concentrations of catechin (AlCl_3_), methionine, and NBT were 1 (0.5), 9, and 0.15 mM in the reactant, respectively. The reactant was kept at pH 7.8 by adding either NaOH or HCl. The reaction solutions were illuminated with blue light at 20 W/m^2^ for 0–60 min. After the photochemical reaction, the proceeding${\;O}_{2}^{•-}$ reduced NBT during the process to form formazan, which was estimated at 560 nm. 

### 4.8. Statistics

The determination of significant differences in each experiment performed in triplicate was achieved via the one-way analysis of variance (ANOVA) method. The unpaired Student’s *t*-test was used when statistical differences were indicated. The results are expressed as the mean ± standard deviation (SD) of each test. The optimum level set for statistical significance was a *p*-value of less than 0.05.

## 5. Conclusions

Catechin is sensitive to blue light and forms a proanthocyanidin by photooxidation under BLI and alkaline conditions. A ferric ion is similar to an aluminum ion in terms of charges and ionic radii. However, an aluminum ion is amphoteric, while a ferric ion is not. Upon the addition of FeCl_3_, catechin turned black via chelation to a ferric ion with or without BLI treatment. In this study, under BLI and alkaline conditions, the addition of AlCl_3_ to catechin inhibited the formation of $O_{2}^{•-},$ and therefore decreased the generation of proanthocyanidin, suggesting that the aluminum superoxide complex was generated via water displacement from the solvated ${\mathrm{Al}}^{3+}$ in an alkaline solution, and the aluminum superoxide complex can enhance electron-transfer processes, leading to proanthocyanidin disconnection. Therefore, as an amphoteric species, aluminum exhibits an inherent potential to stabilize${\;O}_{2}^{•-}$, therefore suppressing catechin photolysis under treatment with BLI in alkaline conditions. Citric acid may enhance the absorption of aluminum in antacids. Lemon tea is a popular beverage, rich in aluminum, catechins, and citric acid. For solutions of catechin in the presence of AlCl_3_ with a large amount of citric acid under treatment with BLI, citrate might compete with molecules containing ${\mathrm{OH}}^{-}$ groups or $O_{2}^{•-}$ from an aluminum superoxide complex. It is therefore of interest to use catechin photolysis as a model for investigating the effects of citric acid on the structural changes of catechin and the alternation of aluminum species under treatment with BLI.
